# From Cookbooks to Networks: A Framework for Comparing Multiethnic Ingredient Systems in Transylvania

**DOI:** 10.3390/foods15061006

**Published:** 2026-03-12

**Authors:** Zsolt Magyari-Sáska, Attila Magyari-Sáska, Lóránt Bálint-Bálint

**Affiliations:** 1 Faculty of Geography, Babes-Bolyai University, 535500 Gheorgheni, Romania; lorant.balint@ubbcluj.ro; 2 Faculty of Mathematics and Computer Science, Babes-Bolyai University, 400084 Cluj-Napoca, Romania; attila.magyari@stud.ubbcluj.ro

**Keywords:** computational gastronomy, ingredient co-occurrence networks, network science, historical cookbooks, Transylvania, food technology, culinary acculturation, community detection, centrality measures, Jaccard similarity

## Abstract

Cookbooks serve as structured records of both ingredient repertoires and the underlying processing logics that define a culture’s culinary identity. By modeling five Transylvanian ethnic traditions—Hungarian, Romanian, Transylvanian Saxon, Jewish, and Armenian—as weighted, undirected co-occurrence networks, we found that interethnic connectivity is driven primarily by technological processes rather than simple ingredient presence. Using purposive sampling, we compiled a harmonized corpus of 1409 recipes and applied explicit ingredient normalization (retention, aggregation, and deconstruction) and a 14-class functional taxonomy. We computed density, clustering, modularity, and centrality measures and compared cuisines with a binary Jaccard index, both at the category level and within four course types. Category networks reveal an exceptionally tight Hungarian–Romanian–Armenian triangle (*J* > 0.95), whereas course-level results show that main dishes exhibit the strongest divergence (*J* < 0.28). These results support a layered identity model of Transylvanian gastronomy: while shared confectionery frameworks in desserts dissolve ethnic boundaries (*M* < 0.17), main dishes actively guard cultural boundaries through distinct technological signatures.

## 1. Introduction

The scientific approach to food culture has undergone a significant methodological transformation over the last decade. Complementing earlier, predominantly qualitative and descriptive studies, the emergence of data mining and network science has given rise to the discipline of ‘computational gastronomy’ [1]. This shift in perspective has yielded significant insights in the analysis of ingredients and recipes: today, recipes are interpreted not merely as cultural documents but as complex, mathematically modelable food data systems. In this context, the network theory framework described by Barabási [2] allows for a quantitative grasp of culinary systems through the lens of ingredient usage, co-occurrence, and structural patterns.

A cornerstone of this field was established by the 2011 study by Ahn et al. [3], which empirically examined the ‘flavor pairing hypothesis’ via the network analysis of flavor compounds. Their results indicated that Western cuisines typically pair ingredients that are chemically similar. By comparison, Jain and colleagues [4] demonstrated during their investigation of Indian cuisine that certain Asian systems often follow a ‘negative food pairing’ principle, where chemically distinct flavors dominate. These studies highlighted that culinary structures are not random; rather, they are organized along stable, recurring structural regularities observable in ingredient selection and combinations. The topological comparison of national cuisines in the works of Caprioli et al. [5] and Tallab and Alrazgan [6] further reinforced the realization that the network patterns of cuisines possess measurable, comparable, and interpretable characteristics.

The aim of this study is to fill this oversight by modeling Transylvanian cuisine as a data-driven network and examining inter-ethnic culinary connections based on recipe ingredient patterns. Initially, we examine whether centuries of co-location have resulted in measurable structural convergence among the cuisines of the region’s ethnic groups (Hungarian, Romanian, Saxon, Jewish, Armenian), or if they instead preserve distinct topological patterns. Building on this, we identify ‘keystone’ elements and ‘gatekeeper’ ingredients that play a prominent role in both structural stability and inter-group connectivity, thereby contributing to an understanding of distinct flavor profiles and ingredient repertoires. Finally, we explore the extent to which these results are category-dependent: does a disaggregated, category-based analysis reveal patterns and discrepancies that aggregated, national cuisine network models might obscure? The core of our argument is that when cuisines exist side-by-side for centuries, their convergence is not just about swapping ingredients. It is about the ‘grammar’ of the kitchen [7], the shared processing technologies that dictate how ingredients are allowed to ‘meet’ in a single dish. We therefore propose an explicit hypothesis: the alignment between these culinary traditions will be much more visible in their shared techniques than in their raw ingredient lists. Practically, this means that while different ethnic groups might keep their own unique flavors, they often adopt a common logic for combining broad food categories. Accordingly, we expect course types governed by widely shared technological bases (soups, desserts) to show higher cross-cuisine similarity than course types where cultural and religious constraints are most salient.

Recipes were selected based on their technological representativeness rather than simple frequency; we prioritized dishes that document the core procedural logic of each community. To minimize temporal noise, the corpus was harmonized within a pre-globalization reference frame (19th to mid-20th century), focusing on sources that act as ‘salvage ethnography’ for linguistic or religious minorities.

Our decision was not to measure cooking techniques directly from step-by-step instructions, primarily because historical sources are notoriously inconsistent in how they annotate the actual process. Instead, we operationalize these technologies indirectly by identifying functional and composite ingredients that serve as technological signatures. Subsequently, we seek to identify evidence of this technology-driven convergence within the network’s architecture, specifically through the emergence of high-centrality hubs and the clear segmentation revealed by modularity and weighted clustering. This allows us to infer the underlying logic of the kitchen even when the written instructions are vague.

The contribution of this research is grounded, on one hand, in the fact that, to our knowledge, this is the first study to analyze Transylvanian (and broadly, Carpathian Basin) gastronomy using the tools of network science on a quantitative basis, moving beyond traditional descriptive ethnographic approaches. On the other hand, the disaggregated, category-based approach represents a methodological innovation that allows for the identification of finer structural differences, offering a reproducible framework for further comparative studies across regions. Ultimately, this approach quantifies ingredient repertoires, technological nodes, and bridging flavoring agents that shape regional food system identity and may inform local sourcing, seasonality, and biocultural diversity discussions.

## 2. Literature Review

Network analysis extends beyond the chemistry of flavors: recipes are also imprints of the standardization of food practices and the transmission of culinary knowledge. As Appadurai [8] argues in his work, cookbooks are the primary media for creating, unifying, and codifying a national cuisine. As such, recipes can be interpreted as ‘edible identity’ [9] and as dynamic carriers of cultural heritage [10,11], while simultaneously recording the repertoire of ingredients, techniques, and categories in a concrete, analysable manner. Matta [12] and Tellström et al. [13] further emphasize how local food culture becomes a tool for representation and branding; yet, these processes cannot be separated from the structural patterns that stabilize ingredient usage and combinations in both everyday and festive dining.

From a methodological standpoint, we can distinguish between studies that use networks mainly as descriptive fingerprints of cuisines and studies that treat networks as an explanatory tool for how culinary rules of combination emerge and persist. Ahnert’s [14] early synthesis positioned computational gastronomy precisely at this boundary: network models can reduce recipe complexity while keeping enough structure to compare cuisines at scale, but they also force explicit choices about normalization, ontology, and what counts as an “ingredient” in the first place. More recently, Herrera’s [15] review shows that the field has moved from foundational pairing studies toward richer, question-driven applications, including cultural explanation, segmentation, and the analysis of recipe evolution developments that directly motivate transparent preprocessing and the interpretive use of modularity, clustering, and centrality in historical corpora.

This cultural dimension is also relevant from a tourism perspective, as gastronomy has become a leading sector in cultural tourism development [16,17]. The presentation of local foods, their underlying narratives, and the culinary heritage of different ethnic groups can enhance a region’s competitiveness and contribute to sustainable tourism [18,19]. Hall and Sharples [20], as well as Scarpato [21], point out that gastronomy is no longer just a supplementary service but has emerged as an independent travel motivation. The modern tourist seeks authenticity [22], attempting to connect with the ‘sense of place’ [23] through food; thus, gastronomy can fundamentally determine the qualitative perception of a destination [24]. On the other hand, the gastronomic experience can also act as a barrier: fear of the unknown and distance from the ‘other’s’ (othering) gastronomy can hinder product development [25,26].

While these cultural and tourism perspectives motivate why recipes matter, our analytical focus is on what recipes encode as food systems data: recurring ingredient repertoires, processing technologies, and stable co-occurrence structures. In this sense, cookbooks function as structured records of ingredient use, culinary techniques, and technological constraints, which can be quantified and compared using network-based models.

Much of the recent progress in the field has been driven by the rise in structured, open-access culinary databases, which have made network-based research far more reproducible. RecipeDB [27] focuses on a curated ingredient structure linked to health attributes, moving the discipline away from ad hoc web scraping and poorly documented preprocessing. Similarly, FlavorDB [28] provides the molecular data needed to ground pairing claims in chemistry, while highlighting how easily “ingredient identity” can become ambiguous without explicit normalization. Ultimately, these resources show that decisions around harmonization (retention, aggregation, or deconstruction) are far more than just technical details; they are substantive methodological choices that define the validity of any culinary inference [29].

The issue of sustainability is also closely linked to the investigation of ingredient and recipe networks. Gössling et al. [30] urge the reduction in tourism’s ‘food footprint,’ the key to which lies in local sourcing and seasonality. Bessière [31] and Pietrykowski [32] highlight the role of traditional foods and the slow food movement in rural development and the preservation of regional identity [33]. In this context, mapping recipe networks holds practical significance: identifying traditional ingredients and technologies can support the strengthening of short supply chains and the protection of biocultural diversity.

Moving beyond simple co-occurrence, a growing body of work now connects ingredients to chemical or even biomedical data through hybrid graphs. In these models, “compatibility” is not just something we observe; it is something we can begin to explain through external descriptors. FlavorGraph [34] is a prime example, linking recipe-scale patterns with compound relations to show how culinary structure emerges from the interplay between statistical tradition and chemical reality. Related projects like HyperFoods [35] extend this to bioactive compounds, demonstrating the potential of network workflows once ingredient identities and technological nodes are consistently encoded—an approach that, while not our primary focus, underscores the broader power of the methods used here [36,37].

While international research extensively documents the network structures of global [38], Indian, or Western European cuisines, Central and Eastern Europe remains underrepresented on the map of computational gastronomy. This gap is also significant in the case of Transylvania (Romania), which, due to its historical attributes, serves as a promising model region for examining the dynamics of multicultural conviviality and identity preservation. Transylvania’s socio-historical uniqueness stems from its centuries-long role as a buffer zone and melting pot between East and West. Kürti’s [39] anthropological research highlights that this ‘remote borderland’ created a social fabric where the permeability of ethnic boundaries was in constant flux. Despite this, gastronomic research on the region has typically been limited to descriptive ethnographic collections or tourism marketing analyses [40]. Although tourism literature has recognized the potential inherent in Transylvania’s multicultural heritage [41,42,43,44], there is a lack of deep structural analysis that would reveal actual interactions between cuisines through quantitative means based on the ingredient structure of recipes.

## 3. Materials and Methods

### 3.1. Data and Preprocessing

We used purposive sampling to build a representative dataset based on the principle of maximum variation: we selected complementary recipe collections that cover the primary source types of Transylvanian culinary culture across both time (19th–21st centuries) and ethnic focus. Our aim was not to statistically represent a hypothetical ‘complete’ Transylvanian recipe canon, but rather to create an analytically representative and reproducible corpus that allows for the reliable examination of ingredient usage patterns and network structures.

When we set out to build the corpus, we made it a priority to include only those recipes that offered a clear, explicit ingredient list and could be assigned consistently to one of our four main categories: appetizers, soups, main dishes, or desserts. We also harmonized the labels across collections, mapping various local subtypes into these four shared domains to ensure we were truly comparing like with like. We selected the following five specific collections, from which 1409 recipes were recorded into a unified data schema:

*Biri Néni Szakácskönyve* (Aunt Biri’s Cookbook) [45]: The first and perhaps most well-known Transylvanian cookbook published in the early 20th century, outlining the core elements of urban Hungarian cooking.

*Două Sute De Rețete Cercate De Bucate, Prăjituri Și Alte Trebi Gospodărești* (200 Tested Recipes for Dishes, Pastries, and Other Household Matters) [46]: The first cookbook printed in Romanian (the original Cyrillic edition was transcribed into Latin script by Ion Drăgușanul). Although the authors’ work includes Western adaptations, we consider it a representative Romanian source for this research because: it is the first systematic record of the region’s culinary terminology in Romanian; it documents the cultural fusion born from the meeting of Phanariot (Greek-Turkish) heritage and Western (French-Austrian) aspirations that shaped modern Romanian national cuisine; and it marks the ‘modernization starting point’ of Romanian gastronomy in contrast to archaic rural traditions.

Issekutz, Sarolta: *Erdélyi Örmény Konyha, Fűszerezve* (Transylvanian Armenian Cuisine, Spiced Up) [47]: Beyond being a simple cookbook, this work functions as a form of gastronomic ‘salvage ethnography,’ conserving the culinary identity of the Armenian community that settled in Transylvania in the 17th century and subsequently assimilated linguistically.

Berecz, Edgár: *Zsidó Hatások Az Erdélyi Magyar Konyhaművészetben* (Jewish Influences in Transylvanian Hungarian Culinary Arts) [48]: This volume documents the Jewish culinary traditions of Transylvania, many of which have largely disappeared. It compiles both the sacred foods of religious holidays and the thrifty dishes of everyday life, demonstrating how strict kosher regulations blended with local flavors. The collection acts as both a culinary guide and a record of a disappearing culture.

Kövi, Pál: *Erdélyi Lakoma* (Transylvanian Feast) [49]: The author, the world-renowned manager of The Four Seasons restaurant in New York, created not a traditional cookbook but a gastro-cultural anthology of Transylvania. Published during the darkest decade of Romanian communism, its declared goal was to document Transylvania’s diverse dining traditions in response to forced homogenization. While the books by Issekutz or Berecz focus on specific ethnic groups, Kövi’s work is the only one that synthesizes all five major gastronomic cultures (Hungarian, Romanian, Transylvanian Saxon, Jewish, Armenian), indicating the specific ethnic affiliation of each dish.

In our research, we consciously excluded contemporary, trend-driven recipe publications and focused primarily on sources documenting 19th and 20th-century culinary logic, essentially pre-dating modern globalization. We chose this period for two main reasons: first, the minimization of ‘noise’ in the network structure stemming from globalization, and second, the management of the retrospective nature common to minority cuisine sources.

Globalization has led to the delocalization of food systems [50], where local ingredients are increasingly replaced by products from global commercial chains. As a result, much of modern gastronomy reflects not only regional traditions but also the offerings of the global food industry and current nutritional trends [51]. According to Trubek [52], contemporary recipe collections often follow a ‘cookable anywhere’ logic, thereby partially detaching from the concept of local character. In network analysis, this can lead to ingredient distortion, as non-place-specific, ‘placeless’ commodities may appear routinely [53].

The chosen period (19th–20th century) represents a transitional era in which numerous regional culinary characteristics appear in a documented, stable form, while the homogenizing effects of global supply chains and fusion cuisine are still limited. Although two sources in our database are bibliographically recent publications [47,48] (Issekutz, 2008; Berecz, 2006), they are primarily retrospective and preservationist in nature: the declared goal of both authors was not to create new recipes but to record the culinary knowledge of a disappearing community. Sarolta Issekutz documented the manuscript legacy and oral tradition of the Transylvanian Armenian community; the recipes in the volume (e.g., angin soup, churut) preserve technologies that have remained stable in community memory and practice over the long term. Edgár Berecz’s work is similarly a reconstruction, compiling the cuisine of Transylvanian Jewry from elderly informants.

Because historical cookbooks are uneven by nature, our goal is not statistical representativeness of all home cooking but analytical representativeness of ingredient-system structure within a pre-globalization reference frame. We therefore prioritize transparent source selection and robustness analyses over exhaustive coverage. Cookbook sizes were not artificially equalized by discarding data. Instead, we retained all eligible recipes and addressed imbalance transparently, reporting recipe counts by cuisine and course (Table 1) and a resampling-based comparability assessment that quantifies how much observed differences can be attributed to sampling variability.

**Figure 1:**
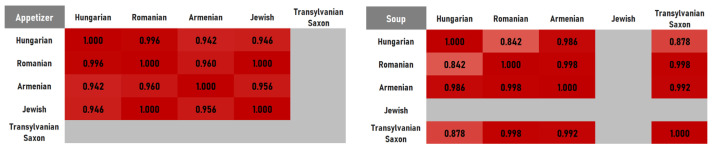

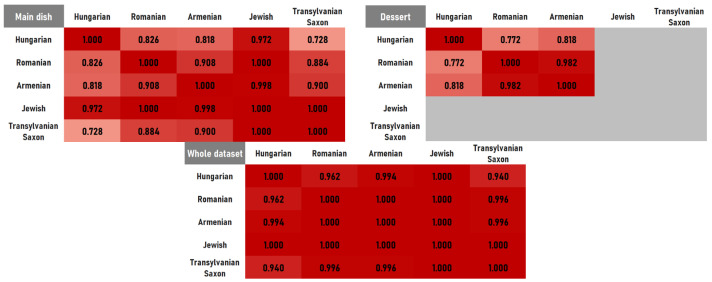
Probabilities that the minimum within-cuisine split-sample Jaccard similarity exceeds the between-cuisine Jaccard similarity for a given cuisine pair.

Because historical recipe corpora are inherently uneven across cuisines and dish categories, we evaluate whether uneven recipe counts compromise cross-cuisine comparisons. We conducted a resampling-based comparability assessment at two levels: pooled cuisine networks built from all dish categories combined and dish-category networks (appetizers, soups, main dishes, desserts). For each cuisine, we generated split-sample stability distributions by repeatedly drawing two independent random subsets of recipes, reconstructing the corresponding ingredient co-occurrence as weighted, undirected networks, and computing the binary Jaccard similarity between the two networks for 500 iterations. Cross-cuisine similarity distributions were obtained analogously by sampling one subset from each of two cuisines and computing the between-cuisine Jaccard. We then quantified comparability for each cuisine pair; the probability that the minimum within-cuisine value exceeds between-cuisine similarity, indicating that sampling variability is smaller than cross-cuisine differences. Dish-category analyses used balanced sampling of 10 per cuisine (where available), while pooled analyses used 33 (the smallest cuisine corpus size). We defined thresholds for reliability, considering comparisons reliably feasible when this value was higher than 0.80, conditionally feasible at 0.60–0.80, and not reliably supported below 0.60. The resulting comparability matrices (Figure 1) show that cuisine-level comparisons remain feasible, supporting the methodological validity of cross-cuisine inference despite uneven historical coverage, except for four dish categories for which the recipe count was below 10.

### 3.2. Network Construction

For each cuisine × course subset, we represent the corpus as an undirected weighted co-occurrence graph G=(V,E,W), where nodes V are ingredients and an edge (i,j)∈E exists if ingredients i and j appear together in at least one recipe. Edge weights are computed as co-occurrence counts across recipes, $w_{ij}=\sum_{r\in R}I(i\in r\wedge j\in r)$, where R is the recipe set and I is the indicator function. Within a recipe, each ingredient is treated as present/absent to avoid spurious weight inflation from textual style.

To sharpen the models, we excluded salt and water, which act as ‘super-hubs’ and would otherwise obscure specific structural differences between national cuisines. Salt’s universal presence—with a weighted degree 16 to 18 times the network average—collapses the topology by drastically shortening average path lengths. Keeping it would mask the finer differences between cuisines, leading to a misleadingly uniform structure. Similarly, the inclusion of water in historical recipes is inconsistent, and despite its high degree of centrality, it carries no distinct organoleptic or flavor information.

During network construction, particular attention was paid to addressing the granularity problem. The raw recipe database contained numerous composite elements; to handle these while ensuring cultural comparability, we employed a three-tiered strategy. The choice of method depended on whether a composite item represented a unique technological signature to be preserved, a functional equivalent to be grouped, or a generic category to be broken down for clarity:

1. Retention: We kept certain composite elements intact where their cultural identity or technological role outweighed their individual components. Examples include churut (which is not merely milk and herbs, but the result of a specific Armenian fermentation process) or roux (a marker of Hungarian thickening technology), as opposed to sour cream thickening.

2. Aggregation: To minimize noise, we merged functionally identical items under common categories—for instance, grouping various dry pastas (macaroni, noodles) by their shared carbohydrate source. We applied a similar approach to “soup stocks” (broth, bouillon) and, in the case of pastries, to “dough sheets” (wafers, puff pastry).

3. Deconstruction: Umbrella terms that would have obscured actual ingredient usage were broken down into their constituent elements. For instance, the category “soup vegetables” (mirepoix/greens) was replaced in the recipes by its specific components (carrot, parsnip, celeriac).

This nuanced data management strategy ensured that the resulting network reflects not only the chemical composition of the dishes but also their cultural and technological structure.

Network analysis was conducted at two granularity levels: the ingredient level and the category level. While the ingredient-level network reveals specific flavor combinations, abstraction to the category level was necessary to filter out noise and reveal the deep structure of national cuisines.

**Figure 2:**
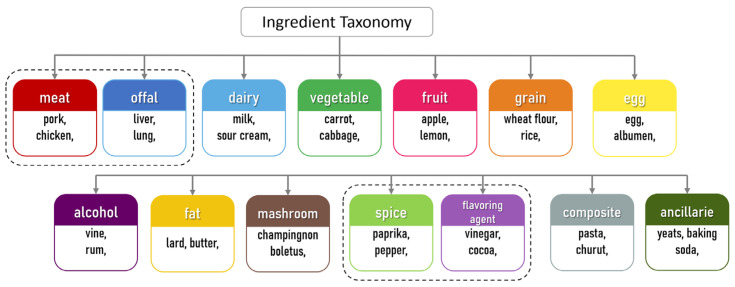
Taxonomy used in network construction and analysis.

For the purpose of this research, we defined 14 main categories (meat, vegetable, grain, dairy, fat, spice, flavoring agent, fruit, egg, mushroom, alcohol, composite, offal, ancillaries). Compared to typical taxonomies, we introduced two important methodological distinctions to accurately capture the specificities of Transylvanian gastronomy: separation of offal and distinction between spices and flavoring agents. We created a distinct category for organ meats (liver, brain, lung, tripe, blood), as the use of offal is a strong socio-cultural marker. In contrast to its marginal role in modern Western diets, in Transylvanian cuisine, the “nose-to-tail” eating culture reflects a historical reliance on culinary resilience in the face of scarcity (Figure 2). We defined spices as dried or fresh aroma carriers of plant origin, while flavoring agents were defined as functional ingredients that determine the fundamental flavor balance of the dish (e.g., vinegar, lemon juice, sugar, honey, mustard, tomato purée). From a network perspective, these two groups behave differently: while spices are often universal and may have high degrees, flavoring agents frequently act as bridges between contrasting flavor profiles.

During network construction, we introduced the composite category to categorize items that are not raw ingredients but complex semi-finished or finished products resulting from technological processes. These composite items function as technological nodes. Breaking them down—such as reducing chocolate to cocoa and sugar—would strip the model of essential preparation data. Maintaining the composite category allows the network to reflect the preparation technology alongside the chemical ingredients. Finally, the ancillaries category includes substances that are neither macronutrients nor primary flavorings but functional chemical agents. These include leavening agents, texture improvers, gelling agents, and preservatives.

### 3.3. Methods

To structurally analyze gastronomic networks and quantitatively compare ethnic culinary traditions, we employ standard network science metrics on the constructed networks.

Weighted degree (*wd_i_*) captures node strength not only for the number of connected neighbors but also for the frequency of these interactions (*w_i_*_,*j*_) [54]. This metric identifies the dominant ingredients. Elements with high weighted degrees constitute the backbone of the cuisine, ranking the core ingredients by their structural prominence.

$${wd}_{i}=\sum_{j=1}^{N}w_{ij}$$

Betweenness centrality (*bc_i_*) measures how often a specific node appears on the shortest path between any two other nodes in the network [55]. This metric identifies ingredients acting as gatekeepers or bridges. These elements connect different dish types or distinct flavor profiles. A spice that is used less frequently but plays a pivotal role in the transition between food categories may possess a high betweenness value. In the analysis of Chinese regional cuisines, Zhu [56] used this metric to identify the regional spices that link the gastronomies of different provinces.

$${bc}_{i}=\sum_{j\neq i\neq k}\frac{\sigma_{j,k}i}{\sigma_{j,k}}$$

where

*i*, *j*, *k*—distinct nodes of the network;

*σ_j_*_,*k*_—number of shortest paths between nodes *j* and *k*;

*σ_j_*_,*k*,_*i*—number of shortest paths between nodes j and k passing through node *i*.

A node’s importance is also determined by the importance of its neighbors [57]. Eigenvector centrality, in turn, gauges what might be termed “gastronomic prestige”. An ingredient receives a high value if it is used alongside other important ingredients. While a side dish might have a high degree, its eigenvector centrality may remain low if it is not connected to complex spice blends.

Ax=λx

where

*A*—adjacency matrix;

*x*—eigenvector centrality vector;

*λ*—eigenvalue of *A*.

Network density (*D*), defined as the ratio of actual edges (*m*) to the number of possible edges based on the node number (*n*) [58], indicates culinary cohesion or conservatism. High density suggests a cuisine operating with a tighter, constant pool of ingredients where everything pairs with everything. Conversely, low density suggests a specialized, segmented cuisine.

$$D=\frac{2m}{n(n-1)}$$

Average clustering coefficient (*C*) represents the probability that two neighbors of a node are also connected to each other, forming triangles in the network [59]. It quantifies the presence of “Flavor Principles” [60]. High clustering indicates that ingredients do not move randomly but in strict groups of three.

$$C=\frac{1}{n}\sum_{i=1}^{n}C_{i}\;\;\;\;\;\;\;\;\;\;\;C_{i}=\frac{2e_{i}}{k_{i}(k_{i}-1)}$$

where

*n*—number of nodes;

*C_i_*—local clustering coefficient;

*k_i_*—degree of node *i*;

*e_i_*—number of edges among the neighbors of *i*.

Modularity (*M*) involves partitioning the network into communities to maximize the density of edges within groups relative to edges between groups [61]. This metric reveals the distinct separation of food groups, and it is often used in culinary networks to measure the topological distance between sweet and savory ingredient clusters [62]. Community detection was performed using the Louvain algorithm [63] for weighted modularity, ensuring that stronger associations contributed proportionally more to the community structure.

$$M=\frac{1}{2m}\sum_{i,j}\left(A_{ij}-\frac{k_{i}k_{j}}{2m}\right)\delta (c_{i},c_{j})$$

where

*m*—number of edges;

*k_i_*—degree of node *i*;

*A_ij_*—adjacency matrix;

*c_i_*—community label for node *i*;

*δ*(*c_i_*,*c_j_*)—same community membership indicator for nodes *i* and *j*.

Jaccard similarity coefficient (*J*(*A*,*B*)) is calculated as the quotient of the intersection and union of two edge sets (*A* and *B*); this comparative metric quantifies cultural overlap. It allows us to determine the extent of similarity in flavor combinatorics between different cuisines. It is applied to investigate cultural diffusion, measuring how much neighboring countries have adopted each other’s recipe structures [64].

$$J\left(A,B\right)=\frac{\left|A\cap B\right|}{\left|A\cup B\right|}$$

**Figure 3:**
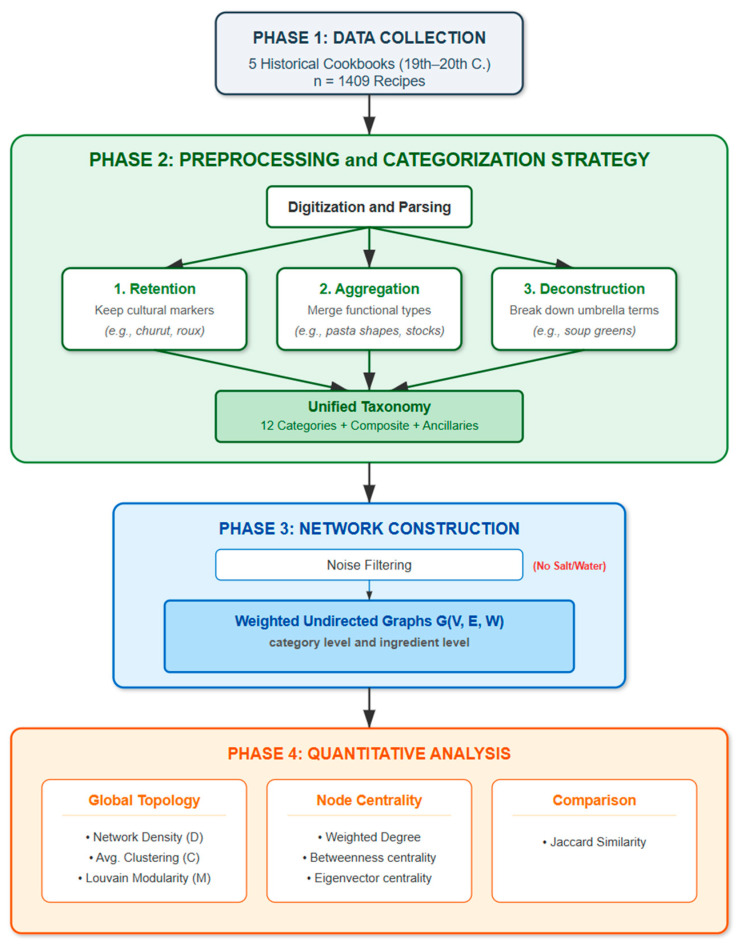
Methodological workflow of the study.

Figure 3 outlines the study’s full workflow, from the initial 1409 recipes through the three-step preprocessing and noise-filtering stages. This pipeline feeds directly into the quantitative framework—covering topology, centrality, and similarity—detailed in the following section.

Finally, to assess the stability of between-cuisine similarity estimates, we implemented two robustness checks focused on the Jaccard overlap of co-occurrence edges. We evaluated internal stability through recipe-level subsampling, recomputing similarities for each replicate (1200 for category-level and 800 for ingredient-level networks) using 80% of recipes without replacement. This allowed us to report a 2.5th–97.5th percentile interval as a consistent stability range. After that, we compared observed similarities to a randomized baseline generated by a within-cuisine ingredient-swap procedure. This randomization preserved recipe sizes and global ingredient frequencies while removing structured co-occurrence, providing a null model (60 realizations per cuisine) against which to measure statistical significance.

The results (Table 2) at the category level, for Romanian, Hungarian, and Armenian cuisine, show very high stability under subsampling (*J* ≈ 0.95). However, our randomization tests revealed that nearly identical overlaps occur even when the data is shuffled (*p* ≈ 1). This suggests that high similarity at this broad level is a general regional baseline. The ingredient level provides a much more specific and reliable signal. For most pairs, the observed similarities consistently exceed what would be expected by pure chance (*p* < 0.02). This confirms that the relationships in the data are driven by deliberate culinary structures rather than just the frequent use of certain ingredients. Similarly, the results for Jewish cuisine proved to be robust, while its overlap with other groups is low; these values are stable and significantly higher than the randomized baseline (*p* < 0.02). This indicates a network shaped by specific internal rules rather than random sampling. Finally, for smaller datasets pairing (Jewish–Transylvanian Saxon), the results did not perform better than the null model (*p* ≈ 0.80). Consequently, we treat these specific cases with caution to ensure the smaller sample sizes do not lead to overinterpretation.

## 4. Results

### 4.1. Category Level Results

**Figure 4:**
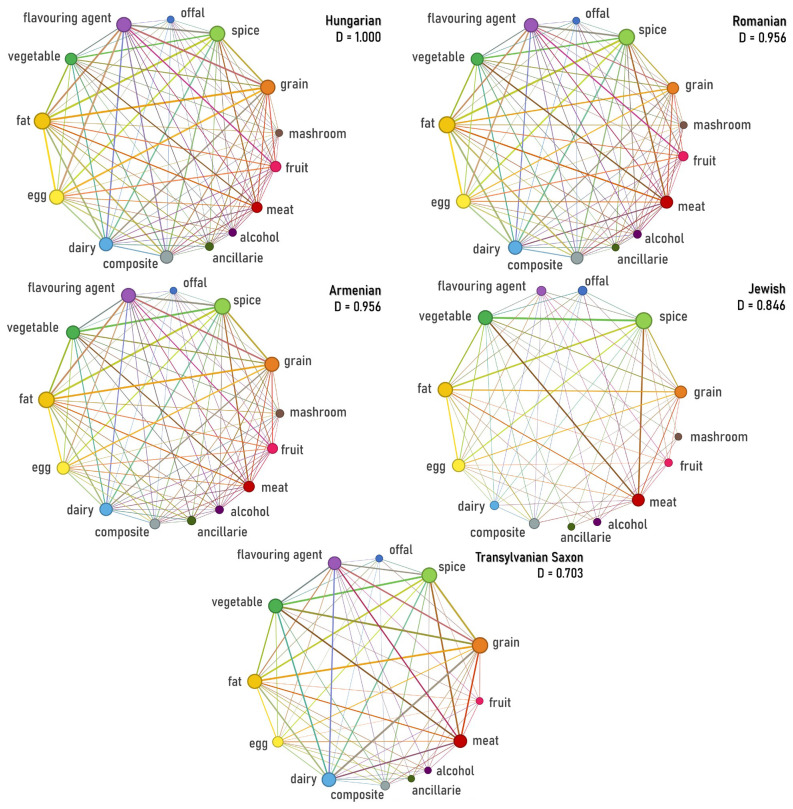
Category-level ingredient co-occurrence networks by cuisine, with corresponding network densities. Node size scales with weighted degrees.

The topological analysis of category-level networks (Figure 4)—node sizes are scaled by weighted degree, and edge thickness represents the frequency of co-occurrence—revealed a structural divergence among the gastronomic systems under investigation. Examination of network density demonstrated that the Hungarian cuisine forms a complete category graph, while Romanian and Armenian networks are near-complete. In practical terms, this means that most category pairs co-occur at least once in our corpus, reflecting a broadly permissive category-level combinatorial space. This stands in sharp contrast to the Transylvanian Saxon and Jewish cuisine networks, which display markedly lower densities (0.846 and 0.703, respectively).

**Figure 5:**
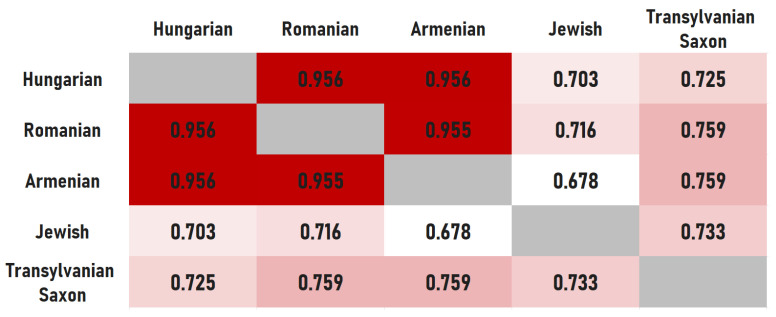
Jaccard similarity index between food category-level networks.

The results obtained from the Jaccard index comparison (Figure 5) map the structural relationships among Transylvanian cuisines. We employed a binary Jaccard index, which focuses only on the presence or absence of edges. This approach removes frequency biases stemming from the varying sizes of recipe collections, focusing strictly on the topological skeleton of the gastronomic systems.

Based on these metrics, the similarity within the Hungarian–Romanian–Armenian triangle is very high (>0.95). This indicates that these three cuisines are highly similar in their category-level edge sets, i.e., they tend to pair the same broad ingredient groups within the 14-class taxonomy. At this aggregate level, the systems are difficult to distinguish because many cuisines in the region share a broad food-group combinatorial baseline. At this level, we observe a few category-level exclusions between these three cuisines; the more discriminative differences emerge at the ingredient-level and in course-specific structures. This near-total alignment implies a functional equivalence of broad food-group roles within the category taxonomy, not an equivalence of ingredient repertoires or recipes. For instance, whether a protein is paired with a root vegetable or a grain, the category-level ‘role’ that each component plays in the meal architecture is often comparable across these three cuisines. This suggests a shared regional standard for what constitutes an acceptable food-group combination at this abstraction level, while leaving room for culturally specific realizations at finer granularity.

Notably, our randomized baselines indicate that near-saturated overlap at the category level can also arise under preserved marginals, reinforcing that this result should be read as a regional baseline rather than as evidence of identical cuisines.

Jewish cuisine shows the least overlap with all other ethnic groups. Several pairings that are common in other cuisines are either absent or extremely rare in the Jewish network, reflecting the practical application of Kosher laws. The lowest similarity value was observed in the Armenian–Jewish relation (0.678). The highest Jewish overlap is with Transylvanian Saxon cuisine (0.733). However, given the smaller Saxon corpus in some course subsets, we treat this as an indicative pattern rather than a definitive statement. Structurally, this overlap may partly reflect that both corpora exhibit fewer cross-category combinations than the Hungarian or Romanian networks at this abstraction level. Specifically, 27 edge types present in the Hungarian network are either absent or negligible in the Jewish cuisine. Among these, the absence of the dairy–meat connection is consistent with Kosher requirements. However, the fruit–dairy connection is also notably weak. This may appear counterintuitive, but it is consistent with the observation that in Transylvanian Jewish recipes, fruits serve primarily as accompaniments to meat dishes or as components of pareve desserts rather than being paired with dairy.

**Figure 6:**
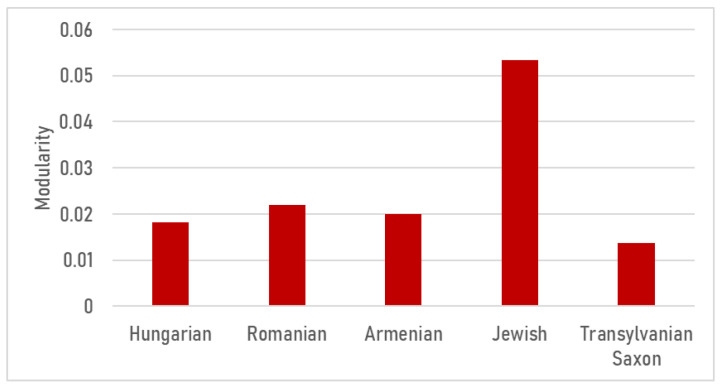
Modularity value for each analyzed cuisine.

Based on the modularity metric (Figure 6), the cuisines suggest three broad groupings. The elevated modularity of the Jewish cuisine network is consistent with the structural effect of kosher dietary constraints: the network fragments into distinct blocks. The nearly identical values observed for the Romanian, Hungarian, and Transylvanian Saxon cuisines reflect a far more cohesive picture, while the Armenian cuisine represents the most compact, fully intertwined structure. In the fragmented Jewish network, neutral (*pareve*) categories—particularly vegetables and grains—emerge as vital bridges due to their high betweenness centrality, standing in sharp contrast to the fully integrated, intermediary-free Hungarian and Armenian networks. In other words, pareve ingredients act as gatekeepers: they carry a disproportionate share of “between-module” connectivity, allowing recipes to traverse the meat–dairy divide without violating kosher rules.

This network architecture suggests a specific ‘bottleneck’ logic: while the Hungarian and Armenian networks are highly redundant and easily navigable through multiple paths, the structural cohesion of Jewish cuisine depends on the stability of a handful of key elements. This configuration is not just a limitation, but a form of culinary optimization. Since the ‘forbidden’ paths between meat and dairy worlds are blocked, the system compresses its flavor information through these permitted ‘gatekeeper’ nodes. This may help explain the high centrality of spices and fats: these elements are not merely flavorings, but the structural ‘glue’ that holds the network together, compensating for the lack of dairy-based thickening technologies in meat dishes. Furthermore, this bottleneck structure implies a unique type of resilience (or fragility) within the system. In the ‘omnivore’ networks of the Hungarian–Romanian triangle, the loss of any single ingredient category could have a minimal impact on overall connectivity. In the Jewish network, however, the removal of certain pareve bridges could effectively cause the culinary system to collapse into isolated islands.

**Figure 7:**
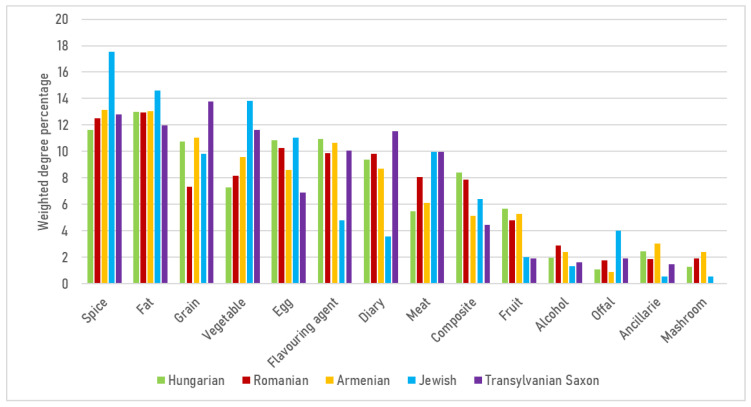
Share of weighted average degree in category-level networks for each analyzed cuisine.

Examining the relative weighted average degree (Figure 7), spices (17.55%) and fats (14.63%) yielded remarkably high values within Jewish cuisine. Since dairy-based thickening agents and the use of sour cream—common staples in Hungarian and Romanian cooking—are prohibited in meat dishes, Jewish cuisine compensates for both flavor and texture through more intensive seasoning and increased fat usage. Jewish cuisine also exhibits the highest proportion of offal (3.99%), reinforcing the “nose-to-tail” philosophy and explaining the prevalence of dishes such as stuffed goose neck, stuffed intestines, and liver-based preparations. While dairy accounts for nearly one-tenth of the network in Transylvanian Saxon (11.55%), Romanian (9.81%), and Hungarian (9.37%) cuisines, this figure drops to 3.59% in the Jewish culinary network. Conversely, Transylvanian Saxon cuisine features the highest proportion of grains. This reflects the central role of pastas (noodles, dumplings) and flour-based dishes, a distinct characteristic of Saxon cuisine.

### 4.2. Ingredient Level Results

Disaggregated analysis revealed that the degree of acculturation varies significantly across different course types. All centrality measures are normalized to [0, 1], and the weighted degree is reported as % of the total strength within the network.

#### 4.2.1. Appetizers

**Figure 8:**
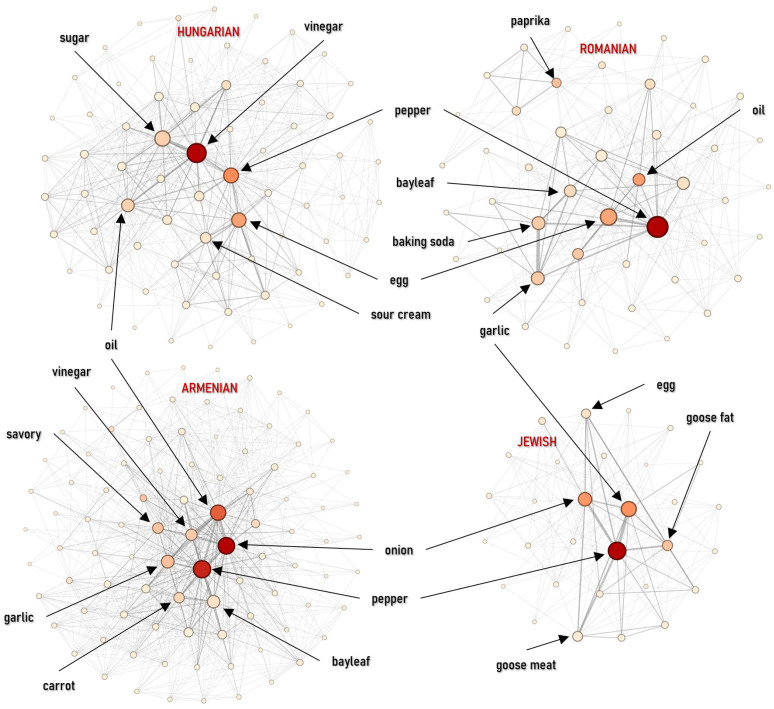
Ingredient co-occurrence network of different national cuisines for appetizers. Node size scales with weighted degree; color intensity indicates betweenness centrality.

**Figure 9:**
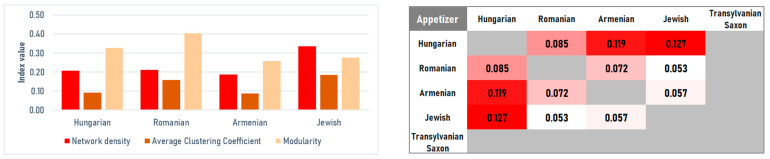
Ingredients co-occurrences network characteristics (**left**) and Jaccard similarity index (**right**) for appetizers.

Appetizer networks differ mainly in how tightly they recombine a limited ingredient set and how strongly they split into distinct sub-styles. The Jewish appetizer network is the most compact and internally reinforced (*D* = 0.3356; *C* = 0.1831), which reflects a closed repertoire where a core set of techniques is reused across many recipes. At the opposite end, the Armenian network is the sparsest (*D* = 0.1865; *C* = 0.0857), pointing to broader ingredient variability and the widest spectrum of ingredients. The strongest internal segmentation appears in the Romanian appetizers (*M* = 0.4024), suggesting multiple well-separated appetizer clusters, such as vegetable spreads versus meat jellies. The Hungarian network is also clearly partitioned but with lower clustering, implying reliance on a few bridging technologies (Figure 8 and Figure 9 (left)).

Centrality metrics reveal distinct flavor profiles driven by specific technologies. In Hungarian appetizers, vinegar is the key organizing element: it leads both in weighted degree (6.8%) and betweenness (0.4686), linking pickling, cold sauces, and salad-like preparations. Jewish appetizers are anchored by an aromatic triad—pepper (11.9%), garlic (10.1%), and onion (8.8%)—while eigenvector centrality also highlights goose fat (0.29) as the technological basis for meat and liver dishes. In the Armenian network, oil stands out in the core (eigenvector 0.35), aligning with a Mediterranean-style reliance on vegetable-based preparations. Romanian appetizers remain spice-centered but show a distinctive core signal for baking soda (eigenvector = 0.31), which serves as a technological marker for the preparation of the traditional mititei (Figure 8).

Although absolute similarity is low across all pairs, the Jaccard Similarity analysis revealed the highest similarity between Hungarian and Jewish cuisines (*J* = 0.1271). This structural proximity likely results from shared preparation technologies for jellies, egg spreads, and pâtés, which bridge the religious divide. Conversely, the structural distance is largest between Romanian and Jewish cuisines (*J* = 0.0541), reflecting the divergence between the Romanian focus on pork and the Jewish focus on poultry (Figure 9, right).

#### 4.2.2. Soups

**Figure 10:**
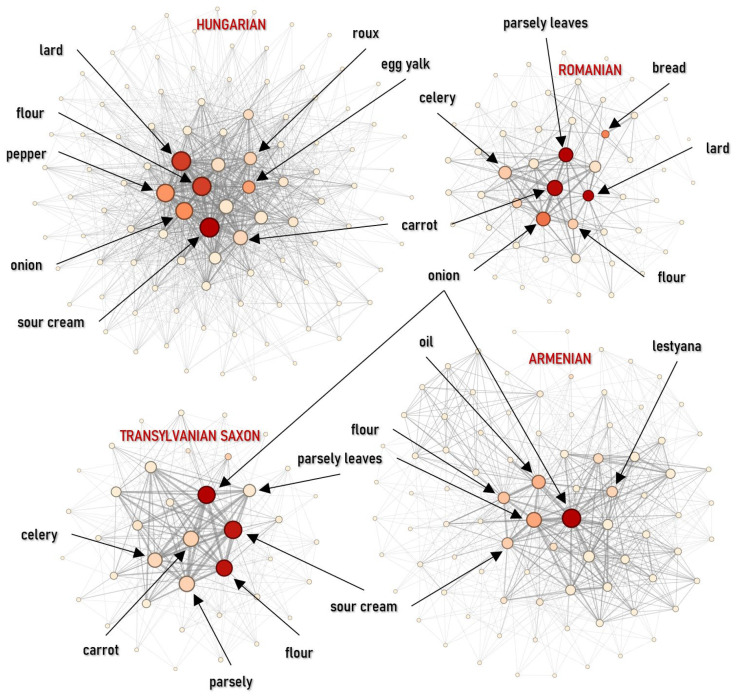
Ingredient co-occurrence network of different national cuisines for soups. Node size scales with weighted degree; color intensity indicates betweenness centrality.

**Figure 11:**
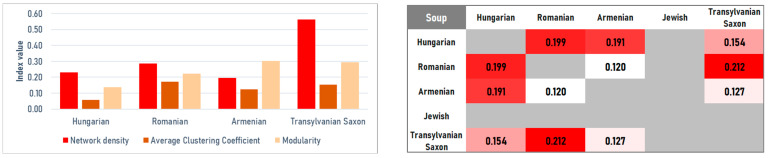
Ingredients co-occurrences network characteristics (**left**) and Jaccard similarity index (**right**) for soups.

The soup networks follow distinct structural models shaped primarily by thickening strategies and souring methods. The Hungarian soup network shows the lowest clustering (*C* = 0.0580) and modularity (*M* = 0.1376), forming a relatively homogeneous structure. This pattern is consistent with the near-universal use of roux and sour cream, which repeatedly links otherwise different soup types. Sour cream functions as the primary bridge (betweenness = 0.2791), supported by lard and flour, indicating that network connectivity is driven more by thickening agents than by the underlying vegetables or meats. In contrast, the Transylvanian Saxon and Romanian networks display markedly higher density (*D* = 0.3821 and *D* = 0.2865, respectively) and clustering (*C* > 0.17), suggesting more rigid ingredient combinations that form recurrent cliques. In the Romanian network, centrality is concentrated on base vegetables—carrots (share 6.87%, eigenvector 0.3681) and parsnips—consistent with a vegetable-forward thickening logic (pulp/stock-based) rather than roux-driven cohesion. The Transylvanian Saxon network, while the densest, is weakly modular (*M* = 0.1202), reflecting a consistent sour-cream/flour model (Figure 10 and Figure 11 (left)).

The Armenian soup network is the sparsest (*D* = 0.1954) yet the most modular (*M* = 0.3028), indicating strong internal segmentation. This likely arises from the use of specialized ingredients such as churut (fermented dairy, eigenvector 0.1874) and distinct herb sets. The Armenian network stands out for its high reliance on onion (share 6.46%; betweenness = 0.4571), which acts as the primary organizing mechanism, supported by oil. This indicates a sautéed aromatic base as the key organizing mechanism, structurally distinct from the Hungarian roux (Figure 10).

The strongest structural proximity in this category (*J* = 0.2123) was found between the Romanian and Transylvanian Saxon traditions. This overlap stems from a shared preference for sour flavor profiles (e.g., vinegar, sauerkraut juice) and the intensive use of root vegetables, producing comparable co-occurrence structures across the two traditions (Figure 11 right).

#### 4.2.3. Main Dishes

**Figure 12:**
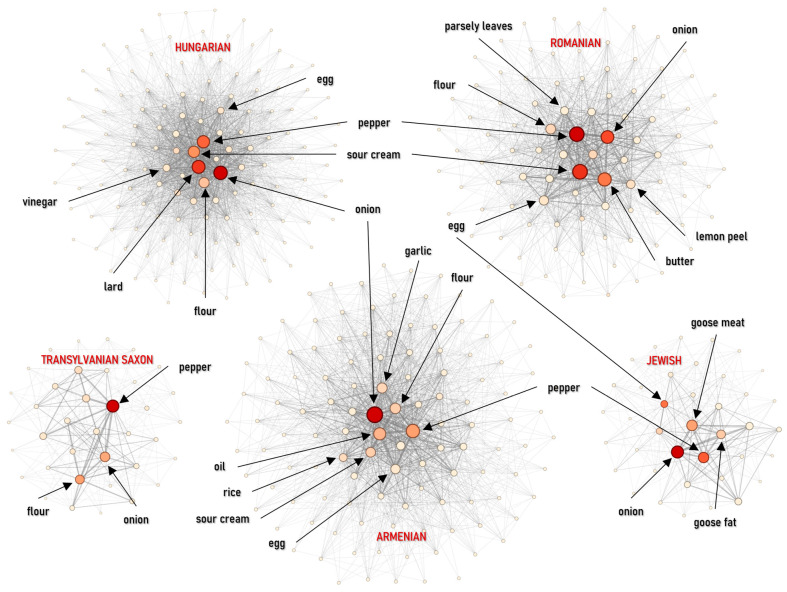
Ingredient co-occurrence network of different national cuisines for main dishes. Node size scales with weighted degree; color intensity indicates betweenness centrality.

**Figure 13:**
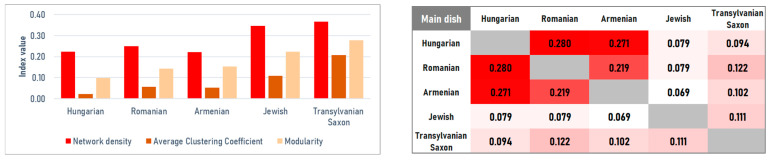
Ingredient co-occurrence network characteristics (**left**) and Jaccard similarity index (**right**) for main dishes.

Structural differences are most apparent in the main course category. The Hungarian main-course network is dense but shows the lowest clustering (*C* = 0.0234) and modularity (*M* = 0.0990), indicating weak internal segmentation and a strongly unified structure. This pattern is consistent with the widespread use of a common technological base—onion (7.05%), lard (6.80%), and pepper (6.35%)—reinforced by a strong thickening layer of sour cream (5.73%) and flour (4.99%). These ingredients link a wide range of dishes, merging the network into a single, homogeneous block where onion acts as the main bridge (betweenness = 0.3793). The Jewish network is also highly dense (*D* = 0.3468) but distinct, reflecting intensive recombination within a limited ingredient pool. Its modularity is higher (*M* = 0.21), likely due to the structural separation between meat and dairy dishes. The core is organized around onion (10.02%), pepper/goose (8.42%), with a clear focus on fats and legumes (goose fat 6.72%, beans 4.90%), consistent with a cuisine-internal technological core shaped by religious constraints on pork. The Armenian mains are more modular (*M* = 0.1527) and show the strongest hinge role for onion (betweenness = 0.4925), supported by an oil–garlic axis (oil 4.81%, garlic 3.76%). Romanian main courses are more internally reinforced and structured (*D* = 0.2494; *C* = 0.0567; *M* = 0.1435). In contrast to the soup category, sour cream (6.53%, eigenvector = 0.3674) and butter (5.75%) lead the centrality rankings alongside pepper (betweenness = 0.3481), pointing to a stronger dependence on creamy, sauce-oriented preparations (tocana) as a structural organizing principle. The Transylvanian Saxon network indicates a more conservative, spice-focused organization: pepper dominance is strongest here, and the combination of high density and clustering suggests consistent variation on a stable core (meat + pepper + onion + flour) (Figure 12 and Figure 13 (left)).

Jaccard analysis indicates that Hungarian–Jewish similarity is among the lowest in the study (*J* = 0.0794), consistent with limited overlap between their dominant technological bases (lard/paprika vs. goose fat/beans). In contrast, the Hungarian–Romanian connection is strong (*J* = 0.2804), and Hungarian–Armenian similarity is also notable (*J* = 0.2708), supported by shared stew-centered technological repertoires (Figure 13 right).

#### 4.2.4. Desserts

**Figure 14:**
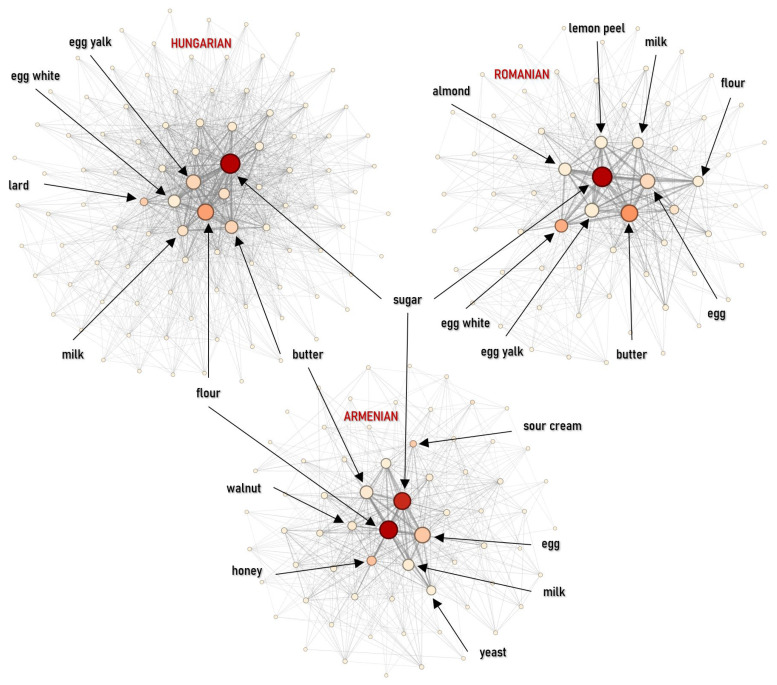
Ingredient co-occurrence network of different national cuisines for desserts. Node size scales with weighted degree; color intensity indicates betweenness centrality.

**Figure 15:**
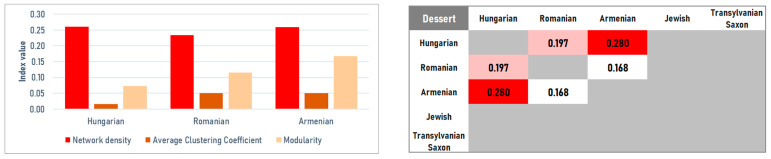
Ingredient co-occurrence network characteristics (**left**) and Jaccard similarity index (**right**) for desserts.

The dessert category shows the highest level of convergence across the cuisines, driven by a shared technological framework—based on a shared sugar–flour–egg–fat core—likely rooted in the confectionery heritage of the Austro–Hungarian Monarchy. Analyzing the three major corpora (Hungarian *n* = 416, Romanian *n* = 92, Armenian *n* = 60), network density is remarkably consistent: Hungarian (*D* = 0.2599) and Armenian (*D* = 0.2592) are nearly identical, while the Romanian network is slightly sparser (*D* = 0.2334). In all cases, modularity remains low (*M* = 0.0728; 0.1676; 0.1153, respectively), indicating limited internal compartmentalization. Instead of forming separate clusters, these desserts form a homogeneous, technology-driven network governed by strict combinations of dough components and sweeteners (Figure 14 and Figure 15 (left)).

Despite this convergence, centrality patterns reveal cuisine-specific signatures. In the Hungarian and Romanian networks, sugar is the primary organizer and main bridge (betweenness: 0.6774 and 0.5855). However, the Romanian profile is distinguished by the prominence of butter (share 9.52%), which outranks flour and eggs, pointing to a stronger orientation toward buttercream-based confectionery traditions. In contrast, the Armenian network places flour at the structural center (share 9.94%, betweenness 0.4111), though it is uniquely distinguished by the use of honey (share 4.13%). This separates the Armenian profile from the purely sugar-centric Hungarian model and suggests a stronger continuity with older, honey-based pastry practices (Figure 14).

The highest structural similarities are found between Hungarian and Armenian dessert networks (*J* = 0.2798). This convergence is likely supported by shared technologies for strudels, braided breads, and complex layered cakes, which generate comparable ingredient co-occurrence structures across the two traditions (Figure 15, right).

## 5. Discussion

Applying network science to gastronomic data moves beyond traditional descriptive food anthropology, offering quantitative evidence on the nature of cultural boundaries and interactions. While culinary identity is often associated with ingredient lists, it resides more deeply in the topology of co-occurrence patterns, a structure that captures both shared kitchen technologies and cultural constraints.

Our findings support a layered model of convergence and separation across both aggregated and course-specific networks. While centuries of co-location produced strong structural convergence for certain groups, the process was not universal, however. At the category level, Hungarian, Romanian, and Armenian networks are nearly indistinguishable (densities 0.956–1.000) and show very high similarity (Jaccard > 0.95). This suggests almost no structural barriers in how these groups combine food categories (Figure 4, Figure 5 and Figure 6).

However, this high-level convergence masks a complex reality revealed only when the data is disaggregated. As we move from broad categories to specific dish types, this uniformity breaks down, particularly in main dishes, where we observe the strongest divergence among all groups (*J* < 0.28). These results support a ‘layered identity’ model of Transylvanian gastronomy. It appears that cultural exchange and boundary maintenance operate at different frequencies depending on the culinary context. Desserts, for instance, function as a shared regional space where ethnic boundaries essentially dissolve (*M* < 0.17). This convergence is largely driven by a shared confectionery framework—sugar, flour, and fats—rooted in the urban baking traditions of the Austro–Hungarian Monarchy.

In sharp contrast, main dishes act as the primary site for ‘boundary guarding’. In this category, ethnic groups maintain distinct technological signatures—specific, rigid ways of combining fats, aromatic bases, and thickening agents—that prevent their culinary identities from merging into a single, undifferentiated network. By comparison, Transylvanian Saxon and especially Jewish networks remain less integrated (densities 0.846 and 0.703), reflecting persistent regulatory and cultural boundaries. This divergence sharpens significantly in course-level networks, where Jewish main dishes form a distinct island despite geographic proximity (Figure 12 and Figure 13).

The keystone elements that stabilize these cuisines and mediate connectivity are predominantly technology-driven. The logic of thickening and souring (roux and sour-cream systems versus vegetable pulp and alternative souring), fermentation markers like churut, and fat systems repeatedly anchor the networks across various cuisines (Figure 7). Centrality patterns highlight, for instance, the onion–lard–paprika triad in Hungarian main dishes and the sour–cream–flour axis in soups as robust, albeit rigid, organizers. In comparison, Romanian networks are more decentralized, with vegetables occupying prominent bridging positions (Figure 8, Figure 9, Figure 10, Figure 11, Figure 12, Figure 13, Figure 14 and Figure 15). In the fragmented Jewish category network, *pareve* ingredients—notably vegetables and grains—act as essential bridges, while intensified seasoning and fat use compensate for prohibited pairings (Figure 6 and Figure 7).

These relationships are highly category-dependent, demonstrating the necessity of disaggregating the data. While a high-level view suggests near-identity within the Hungarian–Romanian–Armenian triangle, course-level networks reveal where boundaries are either guarded or dissolved: main dishes conserve cultural separation (e.g., the very low Hungarian–Jewish similarity), whereas desserts show marked convergence, consistent with the shared confectionery technologies of the region (Figure 12, Figure 13, Figure 14 and Figure 15).

In this framework, culinary technology functions as a primary structuring principle of interethnic coexistence. Our results suggest that convergence in Transylvania did not require the homogenization of ingredient repertoires; instead, it operated through the adoption of shared ‘procedural grammar.’ This common technological platform (the systems of roux, specific fermentation markers, and standardized thickening methods) provided the structural stability necessary for these diverse communities to interact and coexist within the same regional niche. By sharing the ‘how’ of the kitchen while often retaining the ‘what’ (ingredients), these cultures developed a hybrid logic that supported both functional integration and cultural continuity.

Disaggregation also exposes local anomalies, such as the unexpectedly high structural similarity between Hungarian and Jewish appetizers. This pattern is best interpreted as a technological analogy rather than shared flavor identity: frameworks like aspics, pates, and egg-based spreads yield similar network skeletons even when the underlying animal proteins differ (Figure 8 and Figure 9).

Jewish cuisine is not only less connected, but it is connected differently. Because kosher practice restricts direct meat–dairy co-occurrence, the network must route cohesion through neutral ingredients. Vegetables, grains, and eggs repeatedly sit on the shortest paths between otherwise separated recipe clusters, effectively functioning as gatekeepers that preserve versatility under constraint. This mechanism also helps explain why seasoning and fat systems become structurally prominent; they compensate for prohibited pairings by intensifying flavor and texture within the allowed combinatorial space.

It is important to note that the cookbooks used for this analysis are normative sources and do not necessarily reflect the full spectrum of everyday practice. Although their ethnographic authenticity is high, the selection of recipes reflects the collectors’ preferences and historical context. A key challenge in historical computational gastronomy is the inherent unevenness of the corpus. While our resampling-based assessment indicates that the observed network patterns are statistically separable from random noise, we should consider the disparity in recipe counts, most notably the contrast between the extensive Hungarian dessert corpus and the far more limited Transylvanian Saxon and Jewish subsets. Because of this imbalance, we do not present the course-level findings for these smaller subsets as a definitive census of their respective culinary canons. Instead, we interpret them as a structural signal: a high-fidelity snapshot of the underlying logic and technological constraints that governed these kitchens. In these more closed systems, even a smaller number of recipes can reveal a consistent, rule-driven combinatorics, such as the strict avoidance of specific pairings in Jewish cuisine or the repetitive use of flour-based thickening in Saxon soups. However, moving from an indicative signal to a definitive model requires further validation. These results should be viewed as a foundational quantitative framework for Transylvania.

A further limitation lies in the simplification of ingredient coding. The model captures presence, absence, and weighted co-occurrence, but not quantities; therefore, centrality rankings and edge weights approximate structural importance rather than organoleptic intensity. Future work could incorporate quantity information and richer annotations (such as processing steps or seasonality), though historical measurement practices pose a significant challenge.

Network openness (low clustering) and closedness (high density) are not value judgments, but imprints of adaptation strategies. The Hungarian omnivore strategy enables high combinatorial variability, whereas Jewish cuisine optimizes flavor within a strict set of rules, resulting in dense, redundant connections. This study contributes to computational gastronomy by demonstrating that regional cuisines are not homogeneous entities but layered networks: while main courses conserve cultural boundaries, desserts dissolve them. This model of layered identity may explain how multiculturalism persisted in Transylvania for centuries without culinary identities dissolving into a single, undifferentiated network.

## 6. Conclusions

Our findings demonstrate that culinary connectivity in historical Transylvania was structured less by a shared pool of ingredients than by shared technological repertoires. This suggests that co-location alone does not guarantee a melting pot; rather, specific social and religious boundaries can maintain a network’s unique architecture over centuries. While individual flavors often remained distinct, the underlying ‘procedural grammar’—the ways in which food groups are thickened, soured, and combined—provided the common ground for interethnic exchange. Our study proves that network analysis offers a scalable method for tracing these cultural interactions within historical corpora, allowing us to quantify patterns of coexistence that were previously accessible only through qualitative description.

In addition, the study highlights that culinary identity is highly category-dependent. While aggregated national cuisine models often obscure local nuances, a course-level analysis reveals a more complex reality: main dishes tend to guard cultural boundaries, while desserts serve as a shared space for convergence. These findings, including specific anomalies in appetizers, underscore the importance of moving beyond broad generalizations toward a more disaggregated approach.

The study remains constrained by ‘cookbook bias,’ editorial standardization, and the effects of temporal compression across the 19th and 20th centuries. These sources can reflect an idealized culinary canon rather than the full fluidity of everyday practice. To refine this model, future research should integrate archival, ethnographic, and longitudinal data, bridging the gap between printed norms and lived experience. Furthermore, extending this comparative network modeling to other multiethnic regions will be essential to testing the robustness of these patterns and understanding how culinary identities evolve under the pressure of long-term co-location.

## Figures and Tables

**Table 1:** Recipe count for national cuisines by dish category.

	Hungarian	Romanian	Armenian	Jewish	Transylvanian Saxon
Appetizer	33	18	43	11	2
Soup	119	19	34	2	16
Main dish	338	93	68	24	10
Dessert	416	92	60	6	5

**Table 2:** Robustness of cross-cuisine network similarity: subsampling stability (Jaccard) and randomized null baseline at category and ingredient levels.

	Category Level		Ingredient Level	
Cuisine Pair	Jaccard Overlap 95% Range	Null Mean and *p*-Value	Jaccard Overlap 95% Range	Null Mean and *p*-Value
Hungarian–Romanian	0.945–0.978	0.99|1.00	0.257–0.281	0.26|0.02
Hungarian–Armenian	0.912–0.978	0.99|0.98	0.28–0.305	0.29|0.02
Hungarian–Jewish	0.582–0.711	0.76|1.00	0.066–0.092	0.07|0.02
Hungarian–Saxon	0.644–0.733	0.78|1.00	0.104–0.125	0.11|0.02
Romanian–Armenian	0.943–0.966	0.98|1.00	0.200–0.226	0.21|0.02
Romanian–Jewish	0.600–0.716	0.76|0.93	0.075–0.103	0.08|0.02
Romanian–Saxon	0.667–0.759	0.79|0.92	0.150–0.177	0.15|0.02
Armenian–Jewish	0.586–0.693	0.76|1.00	0.066–0.096	0.07|0.02
Armenian–Saxon	0.678–0.776	0.79|0.85	0.106–0.128	0.12|0.16
Jewish–Saxon	0.643–0.818	0.78|0.93	0.120–0.161	0.16|0.80

## Data Availability

The digitized dataset from the cookbooks, including recipe ingredients and their classification, can be accessed at https://github.com/zsmagyari/foodheritage (accesses on 11 March 2026). Recipe names were translated from Hungarian and Romanian.
